# An interview with Kee-Joon Lee

**DOI:** 10.1590/2177-6709.22.4.028-033.int

**Published:** 2017

**Authors:** Amanda Carneiro da Cunha, Eduardo Silveira Ferreira, Lincoln Issamu Nojima, Mariana Marquezan, Matilde da Cunha Gonçalves Nojima

**Affiliations:** 1 » MSc in Orthodontics, Universidade Federal do Rio de Janeiro/RJ. » PhD student in Dentistry (Orthodontics), Universidade Federal do Rio de Janeiro/RJ. » Research Fellow, Department of Orthodontics, Institute of Craniofacial Deformity, Yonsei University/South Korea.; 2 » Associate Professor, Universidade Federal do Rio Grande do Sul (UFRGS), Porto Alegre, Rio Grande do Sul, Brazil. » Professor and Chairman of Orthodontics Department, Universidade Federal do Rio Grande do Sul (UFRGS), Porto Alegre, Rio Grande do Sul, Brazil. » Diplomate of the Brazilian Board of Orthodontics and Facial Orthopedics (BBO). » President-Elect of Association of Diplomates of the Brazilian Board of Orthodontics and Facial Orthopedics (CDBBO). » Fellow of the Associação Gaúcha de Ortodontia (SOGAOR). » Fellow of the Associação Brasileira de Ortodontia e Ortopedia Facial (ABOR). » MSc and PhD in Orthodontics, Universidade Federal do Rio de Janeiro (UFRJ), Rio de Janeiro, Rio de Janeiro, Brazil. » DDS, Pontifícia Universidade Católica do Rio Grande do Sul (PUCRS), Porto Alegre, Rio Grande do Sul, Brazil.; 3 » DDS, Universidade de Passo Fundo/RS. » Specialist in Radiology, Universidade Federal do Rio de Janeiro/RJ. » MSc and PhD in Orthodontics, Universidade Federal do Rio de Janeiro/RJ. » Post-doctoral stage at Case Western Reserve University/OH. » Associate Professor, Department of Orthodontics, Universidade Federal do Rio de Janeiro/RJ. » Visiting Associate Professor, Department of Orthodontics, Case Western Reserve University/OH. » Director, Brazilian Board of Orthodontics.; 4 » MSc and PhD in Orthodontics, Universidade Federal do Rio de Janeiro/RJ. » Post-doctoral stage at Universidade Federal do Rio de Janeiro/RJ. » Associate Professor, Universidade Federal do Santa Maria/RS.; 5 » DDS, Universidade Federal do Rio de Janeiro/RJ. » MSc and PhD in Orthodontics, Universidade Federal do Rio de Janeiro/RJ. » Post-doctoral stage at Case Western Reserve University/OH. » Associate Professor, Department of Orthodontics, Universidade Federal do Rio de Janeiro/RJ. » Visiting Associate Professor, Department of Orthodontics, Case Western Reserve University/OH.

**Figure:**
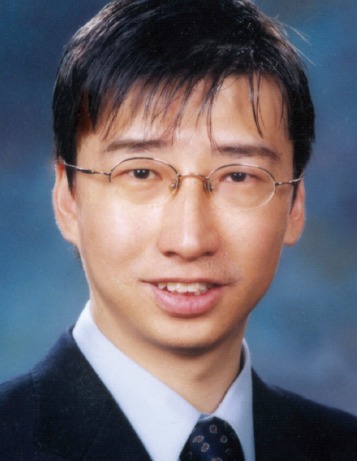

It is a great honor to introduce and conduct the interview with Professor Kee-Joon Lee. I had the opportunity to meet Dr. Kee-Joon in Seoul, in 2015, when we agreed to cooperate in the development of research projects involving the Department of Orthodontics of Yonsei University (Seoul/South Korea) and the Department of Orthodontics of Federal University of Rio de Janeiro (Rio de Janeiro/Brazil). Dr. Lee has world-renowned expertise in biomechanics and miniscrews, which has been published in his numerous articles in the most prestigious scientific journals as well as seen on invitations to lecture in countries worldwide. Dr. Kee-Joon Lee is Professor and Chairman of the Department of Orthodontics of Yonsei University College of Dentistry. He completed the orthodontics specialty training in Yonsei University. He was a visiting scholar at the Department of Biochemistry, University of Pennsylvania School of Dental Medicine in 2002-2004 and at the Division of Plastic Surgery, the Children’s Hospital of Philadelphia in 2010-2011. He was an adjunct professor at the Department of Orthodontics, Temple University, and at the University of Pennsylvania (2010-2011). He has contributed many book chapters on biomechanics of miniscrew-driven orthodontics, non-extraction treatment in adults, up-to-date lingual orthodontic mechanics and surgery-first approached using TADs. He is the first who demonstrated the miniscrew-assisted rapid palatal expander (MARPE) for adults in AJO-DO, which was cited by many other authors. He has published many articles and case reports regarding the treatment of non-eruption, and total arch movement for hyperdivergent face in orthodontic journals including two cover issues in AJO-DO. His research fields include clinical biomechanics regarding TADs application and the suture and bone responses to orthodontic stimulus. He has served as a reviewer in major orthodontic journals, including AJO-DO, *Angle Orthodontists* and *European Journal of Orthodontics*. He has been invited to many international orthodontic conferences around the world. We were honored to receive Professor Kee-Joon Lee last year, in October, for the Meeting of Alumni Association of Graduate Course in Orthodontics of Federal University of Rio de Janeiro. It was one more of his outstanding conferences in biomechanics with miniscrews.

Lincoln I. Nojima - interview coordinator 

É uma grande honra apresentar e conduzir esta entrevista com o professor Kee-Joon Lee. Tive a oportunidade de conhecer o Dr. Kee-Joon em Seul, em 2015, quando decidimos colaborar no desenvolvimento de projetos de pesquisa entre o Departamento de Ortodontia da Universidade de Yonsei (Seul/Coreia do Sul) e o Departamento de Ortodontia da Universidade Federal do Rio de Janeiro (Rio de Janeiro/Brasil). Dr. Lee possui extensos conhecimentos, reconhecidos mundialmente, em biomecânica e na utilização de miniparafusos. Conhecimentos, esses, que estão publicados em numerosos artigos, nas mais prestigiosas revistas científicas, e que se refletem nos inúmeros convites que recebe frequentemente para ministrar palestras em países de todo o mundo. Dr. Kee-Joon Lee é Professor e Chefe do Departamento de Ortodontia da Faculdade de Odontologia da Universidade de Yonsei. Completou sua especialização, mestrado e PhD na Universidade de Yonsei. Foi acadêmico visitante do Departamento de Bioquímica da Faculdade de Medicina Dentária da Universidade da Pensilvânia em 2002-2004 e na Divisão de Cirurgia Plástica, Hospital Infantil da Filadélfia em 2010-2011. Foi professor adjunto do Departamento de Ortodontia, Universidade de Temple, e da Universidade da Pensilvânia (2010-2011). Colaborou com numerosos capítulos de livros sobre biomecânica da Ortodontia suportada por miniparafusos, tratamento sem extrações em adultos, mecânica ortodôntica lingual atualizada e abordagem cirúrgico-ortodôntica envolvendo o uso de dispositivos de ancoragem temporária (DATs). Foi o primeiro a demonstrar o uso do expansor palatino rápido, assistido por miniparafuso (MARPE) para adultos, no *AJO-DO*, tendo sido citado por muitos autores. Atuou como revisor para grandes revistas ortodônticas, incluindo *AJO-DO*, *The Angle Orthodontist* e *European Journal of Orthodontics*. Tem sido convidado para ministrar numerosas conferências sobre temas ortodônticos em todo o mundo. Tivemos a honra de receber o professor Kee-Joon Lee, em outubro de 2016, para o XVIII Encontro dos Ex-alunos Pós-graduados em Ortodontia da Universidade Federal do Rio de Janeiro. Foi mais uma de suas brilhantes conferências sobre biomecânica com miniparafusos.

Lincoln I. Nojima - coordenador da entrevista 

## Regarding the global importance of skeletal anchorage, how do you feel about the effective range of the Yonsei International Mini-residency for Advanced Orthodontics to clinicians worldwide? Matilde Nojima

The mini-residency course in Yonsei University highlights up-to-date clinical orthodontic practices based on sound biological/biomechanical fundamentals that regular orthodontic programs worldwide hardly provides. I think this kind of short and intensive program is effective and important as an essential continuing education program for authorized orthodontists and as an introductory session for dentists who want deep learning about orthodontics.

## Recently in your visit to Brazil, your expertise in the use of skeletal anchorage devices was clearly noticed. What do you highlight as fundamental to the success of orthodontic treatments using orthodontic miniscrews? Eduardo Silveira

Definitely, the biomechanics of intraoral skeletal anchorage means producing constant force regardless of the patients’ compliance. In this context, the treatment outcome is subject to the force system an orthodontist has created. Therefore largely independent mechanics in respective arches (maxillary and mandibular) using miniscrews is recommended instead of combining the miniscrews and intermaxillary elastics, removable appliances and/or extraoral appliances. Any uncertainty caused by those appliances may mask the strength of orthodontic miniscrews.

## With regard to the type of miniscrew used and its insertion technique, do you have preference for self-tapping or self-drilling miniscrews? For insertion procedure, do you use a hand key, manual or motorized contra-angle? Are these choices related to the site of insertion? Mariana Marquezan

I prefer self-drilling miniscrews mainly because of the simple insertion procedure. A pilot drilling take additional time and devices. There is no difference in the success rate either way. Some articles claim lowered success rate in self-tapping technique.

I prefer hand drilling regardless of insertion sites because relatively low rpm, careful monitoring throughout the insertion procedure are important. Every two or three turns I verify the insertion ‘path’ from the occlusal view to make sure that the miniscrew stays off the roots. If an engine is used, a two-hands technique is recommended where the engine driver position is maintained firmly and the insertion path is monitored by dental mirror during the whole insertion procedure.

## Usually after the first surgery technique, the teeth are moved by miniplates. What is your experience with the use of miniscrews in this type of approach? Lincoln Nojima

Most of my surgical patients undergo orthognathic surgery prior to orthodontic decompensation, namely pre-orthodontics orthognathic surgery or surgery first. I use miniscrews because of the versatility of those devices. Intrusion, distalization and/or uprighting of specific tooth/teeth segment is crucial for this procedure and miniplate has limited insertion sites. Additionally, miniplates can hardly be practiced by orthodontists, which is another shortcoming. 

## Facial profile analysis is determinant for obtaining satisfactory clinical outcomes, from both functional and aesthetic perspectives. Regarding a non-surgical approach, what biomechanical strategy should be considered for treating a patient with lip incompetency? Amanda Cunha

A total arch intrusion. This means a dentoalveolar intrusion of entire dentition. The incompetency is regarded as a vertical discrepancy between the soft tissue covering and the underlying hard tissue. Apart from sectional intrusion for gummy smile, for example, reduction of vertical dimension via intrusion of entire dentition is feasible and evidenced by previous studies.

**Figure 1:**
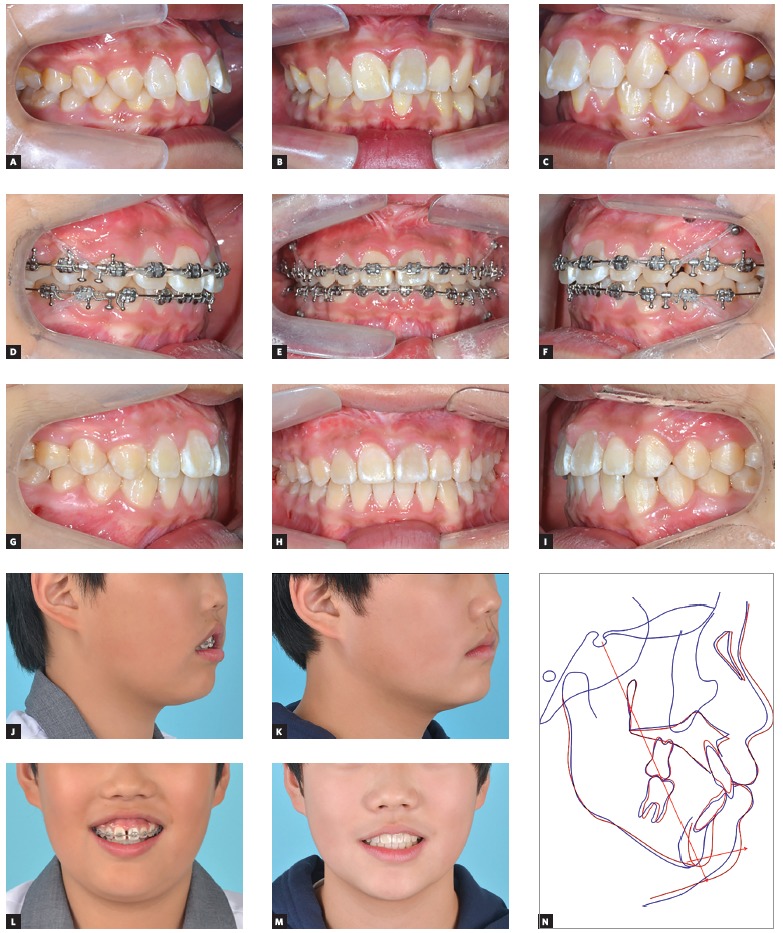
A growing patient (12-year-old boy) showing severe vertical incompetency. A-I) A total arch intrusion was constantly conducted for about 16 months for this growing patient, using full arch bonding and interradicular miniscrews. J-M) Comparison between initial and final extraoral photographs display significant reduction of lip incompetency. N) Cephalometric superimposition reveals the treatment changes in this patient, representing an unusual upward and forward relocation of menton point regardless of the growth.

## What should be considered in the diagnosis of skeletal Class III camouflage cases assisted by miniscrews? Lincoln Nojima

An effective control of the molar and incisor movement. Both require tight translation (bodily movement) to achieve a functionally good Class I molar and esthetically acceptable interincisor relationship. Use of torque in the wire combined with interradicular miniscrews is effective for torque control. A retromolar miniscrew can be used for molar distalization. Anatomic barriers also have to be considered. For example, there is a limitation in mandibular molar distalization due to the lingual cortical plate^1^.

## Regarding the clinical management of impacted teeth cases, what aspects should be evaluated for treating severe maxillary canines impaction in growing patients? Amanda Cunha

Even in case of complicated impaction, rescuing the canine is encouraged because a dental implant at the canine area is highly likely to experience bone recession on the labial side in young adults. Therefore strategic approach is needed to save the tooth. The position of the canine needs to be evaluated first. Then the traction path has to be constructed. This is a typical force-driven approach where force system construction precedes appliance selection. Normally a single force is prescribed but the orthodontist can dictate the position of the button and the direction of extension wire from the attachment to start the traction according to the designed force system. Localization of the presumed center of resistance of canine is important since the tooth normally rotates around the center of resistance in response to a single force. If you do not want this rotation, the line of force should be positioned as close to the center of resistance as possible.

## What factors should be considered as determinant to achieve posttreatment stability in nonsurgical miniscrew-assisted rapid palatal expansion (MARPE) cases? Eduardo Silveira

It is not easy to list the determinants of the stability because it is a multifactorial issue. However, construction of buccolingually upright and tight intermaxillary relationship, i.e., tight interdigitation without tipped molars is an essential factor. One may expect long term stability assisted by natural mastication procedure. Clinically an ‘overexpansion’ up to the upper palatal cusp-lower buccal cusp contact is crucial because of the triangular expansion pattern. This is actually not an overcorrection because at this position, the centers of resistance of respective molars are supposedly aligned along a vertical line. A ‘clinical’ overexpansion followed by natural lingual uprighting during alignment of upper molars using a rectangular wire is a typical procedure using MARPE in adults. Our clinical study reveals around 10% relapse over three years follow-up.^2^ Personally I claim the expansion in adults are doable in the sense of feasibility and stability.

## How is your experience in the clinical management of MARPE technique in growing patients? What is your opinion about the benefits of this approach at these age? Matilde Nojima

Even in growers complete maintenance of expansion during consolidation period is essential. Some bone dehiscence following consolidation phase has been reported previously. I do use MARPE in growers if the arch constriction and/or space deficiency is severe. I also use MARPE in repeated expansion cases. One should always consider this as a ‘distractor’ and the maintenance of sutural gap during consolidation using a tooth-borne appliance in theory is not guaranteed. In this context, the use of MARPE in growers in more than recommended.

**Figure 2:**
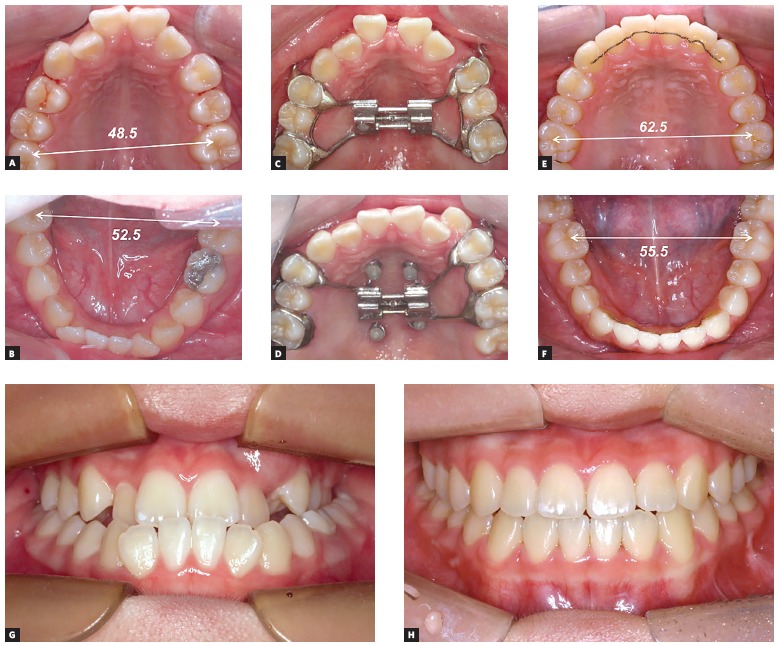
An adolescent case (12 year-old female) with rapid palatal expansion (RPE) followed by MARPE to secure arch expansion. To resolve space deficiency and transverse problem (A, B), a repeated expansion (firstly with conventional [C] and secondly with MARPE [D]) was performed due to the relapse during treatment. Following the occlusal settling, a total of 14.0 mm expansion in the maxilla was gained, compared to the minimal growth change in the mandible (E, F). A significant change in the occlusion is demonstrated: before (G) and after (H) treatment, without surgical intervention.

## How is the acceptance of adult patients and their reports of pain and discomfort when submitted to MARPE? What are the recommendations for relieving painful symptoms? Mariana Marquezan

Very few patients has so far claimed pain or discomfort. Pain is not necessarily the symptom even in adults. Some discomfort may be there due to the bulk of the appliance but very soon most patients get used to it. Apart from pain, a vigorous brushing on deep palate is regularly instructed to prevent the mucosal swelling that may lead to secondary inflammation. 
